# Multi-field query expansion is effective for biomedical dataset retrieval

**DOI:** 10.1093/database/bax062

**Published:** 2017-09-07

**Authors:** Mohamed Reda Bouadjenek, Karin Verspoor

**Affiliations:** 1 School of Computing and Information Systems, The University of Melbourne, Parkville, VIC, 3010, Australia

## Abstract

In the context of the bioCADDIE challenge addressing information retrieval of biomedical datasets, we propose a method for retrieval of biomedical data sets with heterogenous schemas through query reformulation. In particular, the method proposed transforms the initial query into a multi-field query that is then enriched with terms that are likely to occur in the relevant datasets. We compare and evaluate two query expansion strategies, one based on the Rocchio method and another based on a biomedical lexicon. We then perform a comprehensive comparative evaluation of our method on the bioCADDIE dataset collection for biomedical retrieval. We demonstrate the effectiveness of our multi-field query method compared to two baselines, with MAP improved from 0.2171 and 0.2669 to 0.2996. We also show the benefits of query expansion, where the Rocchio expanstion method improves the MAP for our two baselines from 0.2171 and 0.2669 to 0.335. We show that the Rocchio query expansion method slightly outperforms the one based on the biomedical lexicon as a source of terms, with an improvement of roughly 3% for MAP. However, the query expansion method based on the biomedical lexicon is much less resource intensive since it does not require computation of any relevance feedback set or any initial execution of the query. Hence, in term of trade-off between efficiency, execution time and retrieval accuracy, we argue that the query expansion method based on the biomedical lexicon offers the best performance for a prototype biomedical data search engine intended to be used at a large scale. In the official bioCADDIE challenge results, although our approach is ranked seventh in terms of the infNDCG evaluation metric, it ranks second in term of P@10 and NDCG. Hence, the method proposed here provides overall good retrieval performance in relation to the approaches of other competitors. Consequently, the observations made in this paper should benefit the development of a Data Discovery Index prototype or the improvement of the existing one.

## Introduction

Biomedical data include large datasets, with diverse types of information, that are managed by a wide range of biomedical research centers. The data range from medical imaging to bioinformatics sequences, and include research articles, clinical trials, proteomic data, etc. A list of 64 repositories providing such biomedical data have been integrated in the DataMed project by the bioCADDIE team (1,2) (https://datamed.org/repository_list.php). This represents a huge amount of heterogeneous data, primarily used in research projects to achieve a variety of goals, including drug discovery, disease study, gene identification, etc. It is valuable to supply researchers with a unified platform for aggregating, searching and retrieving these biomedical data according to their needs.

However, knowing that these data are heterogeneous, semi-structured, provided by different research centers with different schemas, and are variably annotated with metadata, it is a challenge to develop an aggregation platform for searching biomedical data, in terms of data integration and information retrieval. Hence, we propose in this paper a method for retrieving and searching biomedical data with heterogenous schemas through query formulation.

A typical dataset available in, for instance, the gene expression repositories may contain a description, a list of keywords, and a list of organisms. A typical dataset available in the protein structure repositories contains, in addition, a list of genes and a list of research articles. Hence, the dataset schemas may vary from one repository to another. Hence, inspired by the evaluation framework of the bioCADDIE challenge (2) (https://biocaddie.org/biocaddie-2016-dataset-retrieval-challenge), in this work, we consider only the fields that we believe are the most relevant fields for that task. These fields include (1) title, (2) description, (3) a list of keywords, (4) a list of organisms, (5) the titles of the associated research articles, (6) the abstracts of the associated research articles, (7) a list of genes, (8) a description of a disease and (9) a description of a treatment. These fields are explored in detail this paper to show the importance of each for obtaining the best retrieval performance.

**Figure 1.**
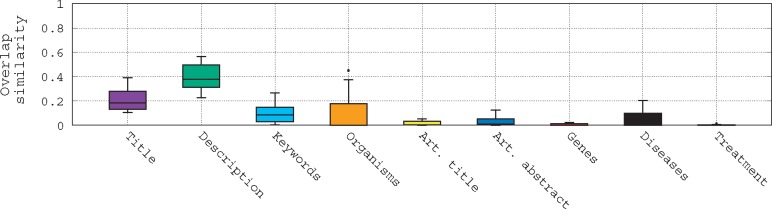
Box-plots of the overlap similarity between the queries and different fields of their associated relevant datasets.

To assess the difficulty of querying such biomedical data, we refer to Figure 1. This figure shows the term overlap similarity (We use the overlap similarity to emphasize the number of terms of a query that are in its relevant datasets. Here, $Overlap(X_{1},X_{2})=\left|X_{1}\cap X_{2}\right|/min(\left|X_{1}\right|,\left|X_{2}\right|)$.) distribution between the queries and different fields of their associated relevant datasets given in the bioCADDIE challenge using box-plots. Several trends can be observed here: (i) first, most of the fields of the relevant datasets have very low term similarity with the queries; (ii) second, the highest similarity with the queries is observed with the description field followed by the title field; (iii) third, even with the description field, the median is still low (<0.4); and (iv) finally, for the genes and treatment fields, there is almost no overlap similarity with the queries, even if some queries do mention genes and diseases. This shows clearly that the queries are quite general, and lack specificity to individual database records, posing a challenge for retrieval of the most relevant datasets.

Given the observed low overlap similarity in Figure 1, we suggest an investigation of query formulation (3) methods as a means for improving the term overlap between queries and relevant datasets. This query formulation includes identifying the correct terms in a query to search specific fields using a multi-field query strategy, and then enriching the multi-field query through a query expansion process. We compare and evaluate two query expansion strategies, one based on the Rocchio method and another based on a biomedical lexicon. We demonstrate the effectiveness of our multi-field query method compared to two baselines, with MAP improved from 0.2171 and 0.2669 to 0.2996. We also show the benefits of query expansion, where the Rocchio method allows us to improve the MAP for our two baselines from 0.2171 and 0.2669 to 0.335.

In summary, in the purpose of building the best queries to retrieve the most relevant datasets, we seek to answer the following questions:

- What parts of the datasets should we query to achieve an effective search in terms of retrieval performance?
- Which method works the best for query expansion, and in which settings?
- What is the best source for term expansion? Do fields of datasets serve as better sources of expansion terms, or is an external biomedical lexicon a useful resource?

To answer these questions, we perform a thorough analysis of query formulation for biomedical search in the context of the bioCADDIE challenge.

This paper presents exactly the system runs we have submitted to the bioCADDIE Dataset Retrieval Challenge 2016, except that one run for the original challenge was built using manual query construction with a biomedical lexicon, and we present an automated version of that run in this paper. Details are given later in the paper.

The rest of this paper is organized as follows: in the next section, we discuss the related work; then, we describe the bioCADDIE challenge dataset collection; next, we provide an architectural overview of our solution followed by the query expansion strategy we use; then, we present the experimental evaluation, followed by a discussion and a summary of key observations.

## Related work

There is a substantial body of research related to query reformulation in the context of IR. Previous research has focused mainly on query expansion and query reduction, as reviewed below.

### Query expansion

Query expansion has been widely explored in the IR literature and in various contexts (4). Hence, we will not review all existing works but we simply provide an insight into what has been done. We review the existing works based on the data sources used to extract relevant terms to enrich the initial query.

A first type of data source that has been used in query expansion methods relies on external lexical semantic resources, typically dictionaries, thesauri or other similar knowledge representation sources such as WordNet (5). For example, Fang (6) has demonstrated that global expansion based on WordNet and co-occurrence based resources can lead to performance improvement in an axiomatic model of information retrieval. Similarly, Mahdabi et al. (7) demonstrated the value of using a patent lexicon for query expansion in the context of patent search. Also, Xu et al. (8) proposed to explore the use of Wikipedia as an entity repository as well as its internal structure for query expansion; they show the effectiveness of this query-dependent approach to improve over a baseline relevance model.

In several common cases, terms for query expansion are obtained from within the data source (typically, the document collection itself). Terms are extracted using a Pseudo-Relevance Feedback (PRF) set of top-*k* ranked documents obtained usually after an initial execution of the query. The Rocchio method (9,10) described later in this document is the best known method of this type. Several methods based on a PRF set have been described and evaluated [e.g. (11–15)]. In the same vein, several approaches are based on preprocessing top retrieved documents for filtering out irrelevant features prior to the utilization of a term-ranking function (4). Hence, several methods for finding more compact and informative document representations have been proposed, such as passage extraction (14) and text summarization (16). In Chang et al. (17), the document summaries go through a further process of clustering and classification with the aim of finding an even more reduced set of orthogonal features describing each document (termed query concepts).

Several research works explored the use of the vocabulary used in social networks as sources of terms for query expansion (18–21). Almost all these approaches model the social network vocabulary as a structured lexicon, and the most related terms are selected by some method to enrich the initial query. A number of these approaches are reviewed in (22).

Since query logs are a rich source of information about users, they have been used for many tasks, one of which is query expansion (23–27). Much of the previous work operates at the level of the whole query, which is more commonly known as query recommendation. This class of techniques first clusters similar queries based on commonly clicked documents (23,26) or the similarity of vocabulary used in clicked documents (27), and uses queries in the same cluster as recommendations for one another.

Finally, in the case of lack of availability of query logs, others researchers proposed to use anchor text (Anchor text is the clickable text in a hyperlink.) to simulate the important parts of a log (28–30). For example, Kraft and Zien (29) demonstrated that using anchor text to refine a query works better than using the full document collection itself.

### Query reduction

While it is known that query length in operational interactive IR systems (in particular Web search engines) is rather short, typically between two and three terms long (31,32), in other IR contexts queries may be longer, ranging from ten to thousands of terms (33,34). Therefore, several researchers have investigated strategies for query length reduction, for the purpose of removing ambiguous and noisy terms.

Kumaran and Carvalho (34) developed an automatic method for reducing long TREC description queries. Using query quality predictors such as Clarity (35), they converted the query reduction task into a problem of ranking reduced sub-queries based on their predicted effectiveness. Their results on TREC Robust 2004 showed the utility of automatic query reduction. This idea has been further enhanced by Arguello et al. (36), who proposed the following procedures: (1) generate a candidate set of sub-queries to consider, (2) predict the retrieval performance of each candidate sub-query and (3) combine the retrievals from the top-k sub-queries with the highest predicted performance. Also, Balasubramanian et al. (37) also studied how to improve performance by reducing queries using quality predictors; however, their system only removes up to one term from the query. This approach is not viable when dealing with very long and descriptive queries. Xue et al. (38) focused on reducing TREC topic descriptions and trained a sequential model to label each queryterm as ‘seryt or ‘re not keepy using query performance predictors as features in a Conditional Random Field model. The authors found greater improvements by combining the predicted sub-query with the original. Xue and Croft extended this idea in (39) by combining sub-queries in a weighted fashion, setting the mixing parameters based on the LTR output. Zhao and Callan (40) trained a classifier to predict a query termyc importance by combining performance predictors with features such as the query-term’s rareness, abstractness, and ambiguity. Their results found greater improvements for more verbose queries (i.e. TREC descriptions vs. v titles).

In the context of patent prior art search which involves finding previously granted patents that may be relevant to a new patent application, the query is often an entire document with hundreds or thousands of words organized into several sections. In that context, the length of the queries led several research groups to investigate query reduction. In (41), the authors proposed a query reduction technique, which decomposes a query (a patent section) into constituent text segments and computes Language Model (LM) similarities by calculating the probability of generating each segment from the top ranked documents (PRF set). Then, the query is reduced by removing the least similar segments to the query. In (33), the authors proposed to study several query reduction techniques and their impact on the task of patent prior art search. They have shown that while query reduction techniques have a mitigated impact on mid-length queries, they are very effective on long queries such as an extended abstract or a description. Also, in (42), authors have shown that a simple and minimal interactive relevance feedback approach outperforms the best result from the CLEF-IP 2010 challenge (43), which was a sophisticated and very advanced system that utilized a very important feature of patents. This suggested the promise of interactive methods for term selection in patent prior art search. For query reduction in medical search, Luo et al. (44) built a search engine that performs query reduction by filtering non-important terms based on their tf-idf score. Their system is designed for lay people performing health search on the Web and does not focus on medical literature retrieval. Also, Soldaini et al. (45) studied the impact of query expansion and reduction methods that take advantage of medical domain knowledge, as well as general purpose IR techniques. Then, they proposed an approach that combines both methods, and which achieved a statistically significant improvement.

However, as the test queries provided are short with an average of 12 terms, we have decided to not consider query reduction in our experiments. Hence, in this paper, we consider query reformulation as the transformation of the initial query to a multi-field query, and then explore the application of query expansion techniques.

## Description of the bioCADDIE dataset collection

The collection of the bioCADDIE challenge contains structured and unstructured metadata from a set of 20 individual repositories (1) (https://biocaddie.org/biocaddie-2016-dataset-retrieval-challenge). The collection contains a total of 794 992 datasets that was frozen from the DataMed backend on 24 March 2016, which are provided in both XML and JSON format. Each dataset contains three main parts: (i) *DocID*, which contains a unique number to identify the dataset in the collection; (ii) *TITLE*, which gives a short summary of the dataset; and (iii) *METADATA*, which provides a set of useful information related to that dataset. These three parts given in an XML format are common for all the datasets. However, given the fact that the whole collection is built through the integration of 20 different repositories, the internal structure of the metadata section varies, and is specific for each repository. Hence, we have implemented a specific parser for each repository to extract the information provided within the metadata section. Table 1 provides details of the information extracted from the collection according to each repository. Table 1 shows clearly the heterogeneous nature of the collection; the information given in the metadata sections is different for each repository. For example, the *genes* section is only available in the *PDB* (46) (The Protein Data Bank (PDB) archive is the single worldwide repository of information about the 3 D structures of large biological molecules, including proteins and nucleic acids.) repository, whereas the *treatment* section is only available in the *ClinicalTrials* (47) (ClinicalTrials.gov is a registry and results database of publicly and privately supported clinical studies of human participants conducted around the world.), *NeuroMorpho* (48) (NeuroMorpho.Org is a centrally curated inventory of digitally reconstructed neurons associated with peer-reviewed publications.) and the *PeptideAtlas* (49) (PeptideAtlas is a multi-organism, publicly accessible compendium of peptides identified in a large set of tandem mass spectrometry proteomics experiments.) repositories. Moreover, as the data collection is an integration of heterogeneous repositories with fundamentally different data, they are grouped by category as shown in Table 1 (https://datamed.org/datatypes.php).

**Table 1.** bioCADDIE dataset collection details

Category		Repository	DocID	Title	Metadata							Total
					Description	Keywords	Organisms	PMID	Genes	Diseases	Treatment	
Clinical trials	1	ClinicalTrials	x	x	x	x	—	—	—	x	x	192 500
	2	CTN	x	x	x	x	x	—	—	—	—	46
Gene expression	3	ArrayExpress	x	x	x	—	x	—	—	—	—	60 881
	4	GEMMA	x	x	x	—	x	—	—	—	—	2285
	5	GEO	x	x	x	—	x	—	—	—	—	105 033
	6	Nursadatasets	x	x	x	x	x	—	—	—	—	389
Imaging data	7	CVRG	x	x	x	—	—	—	—	—	—	29
	8	NeuroMorpho	x	x	—	—	x	—	—	—	x	34 082
	9	CIA	x	x	—	—	x	—	—	x	—	63
	10	OpenFMRI	x	x	x	—	x	—	—	—	—	36
Phenotype	11	MPD	x	x	x	—	x	—	—	—	—	235
	12	PhenoDisco	x	x	x	—	—	—	—	x	—	429
Physiological signals	13	PhysioBank	x	x	x	—	—	—	—	—	—	70
	14	YPED	x	x	x	—	x	x	—	—	—	21
Protein structure	15	PDB	x	x	x	x	x	x	x	—	—	113 493
Proteomic data	16	PeptideAtlas	x	x	x	—	x	x	—	—	x	76
	17	Proteom Exchange	x	x	—	x	x	—	—	—	—	1716
Unspecified	18	BioProject	x	x	x	x	x	—	—	—	—	155 850
	19	Dataverse	x	x	x	—	—	—	—	—	—	60 303
	20	Dryad	x	x	x	x	—	—	—	—	—	67 455

	*Total*			794 992	759 131	531 449	474 206	113 590	113 493	192 992	226 658	

(x) means the information is provided, (—) means the information is not provided. These marks do not imply any ‘positive’ or ‘negative’ information except for the presence or the absence of the considered information in the metadata section.

Regarding the PMIDs (PMID is the unique identifier number used in PubMed.) obtained from the datasets of the *PDB*, *PeptideAtlas*, and the *YPED* (50) (The Yale Protein Expression Database (YPED) is an open source system for storage, retrieval, and integrated analysis of large amounts of data from high throughput proteomic technologies.) repositories, we retrieved the corresponding research articles using MEDLINE to gather additional information about these datasets. MEDLINE is a bibliographic database, which contains 27 million references for biomedical literature publications, and contains mainly the Title and the Abstract of each research article.

A training set was provided for parameter tuning, which includes six queries with retrieved results for which the relevance judgments have been annotated. As for the test set given for the challenge, it consists of 15 queries derived from instantiations of competency questions from three use cases collected from various sources as a part of the bioCADDIE project [for more details on construction of the collection see (2)].

Given this small number of queries in the test set compared with other IR tasks, one can think that any method developed on the top of this test set may overfit especially during parameter tuning. However, given the diversity of the queries, we believe that this should not be the case. Indeed, the test queries given for the challenge span different query types including: the search for genes, the search for particular diseases, the search for proteomic data, the search for gene expression data, etc. The test queries are described in Table A5. As for the relevance judgments (often referred to as qrels—query relevance set) composed of a list of qid/docid pairs, detailing the relevance of documents to topics, it consists into 142 805 datasets judged using the following relevance degree scales: (2) relevant, (1) partially relevant, (0) irrelevant and (−1) unjudged.

## Architecture overview

**Figure 2.**
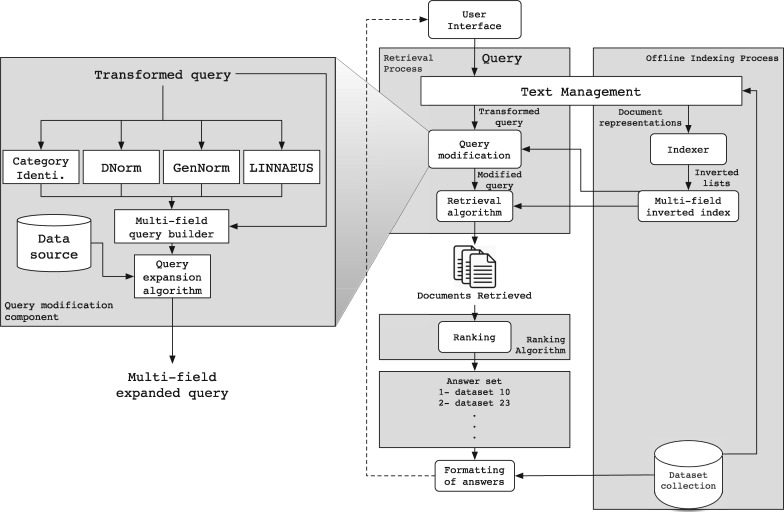
Architecture overview.

The IR system we have developed to tackle the bioCADDIE retrieval challenge is mainly based on the Lucene 6.4.0 IR System (http://lucene.apache.org/). In particular, we re-used the multi-field indexing engine of Lucene, which allows indexing of individual fields associated with documents separately, such that each field can be queried independently (title, description, keyword, etc.). We also re-used the query-document matching mechanism provided in Lucene, which allows retrieval of documents that match the query. Finally, we reused the standard Lucene cosine-based similarity function to score and rank documents accordingly. Figure 2 shows the architecture of our solution, which is composed of two complementary sub-processes, an off-line and an online sub-process. We briefly describe each below.

### Off-line sub-process

As illustrated in the right part of Figure 2, the *Text Management* component parses each dataset in the collection according to its repository structure to extract its fields (title, description, keyword, etc.). Next, each field is processed by reducing its full set of words to a set of tokens by (i) removing the stop words such as articles and connections, and (ii) reducing distinct words to their common grammatical root by applying a stemming algorithm. Then, the *Indexer* component applies a specific organization to the whole extracted content for each field (section), to create an inverted index per field over the collection of datasets. This indexing strategy allows us to query each field separately, and thus, allows boosting of fields in the retrieval algorithm. Finally, the index obtained contains 11 fields that we use in the rest of this paper.

### On-line sub-process

This sub-process handles the userle query as illustrated in the left part of Figure 2. The query is generally provided in the form of keyword query or question, and is reduced by the *query-processing* engine following the same strategy applied to source repository data (except that no fields are available to be extracted in a query). The resultant set of index terms extracted from the user query is then transformed through the *Query expansion & Modification* module.

First, as illustrated in Figure 2, this module processes the query in parallel with four sub-components, *Category Identification*, *DNorm* (51), *GenNorm* (52) and *Linnaeus* (53). The category identification tools identifies what type of data are targeted by the query, and the other tools tag mentions of biological entities including diseases, genes and organisms using natural language processing methods. The category identification step uses a simple rule-based approach to relate specific keywords to the type of data targeted (gene expression, clinical trials, protein structure, etc.). This includes key phrases such as ‘search gene expression’, ‘search protein sequencing’ and ‘search proteomic data’. Each such phrase is associated with a certain data type, and the entity types are also related to specific data fields. After this analysis, the resultant tagged query is then processed by the *Multi-field query builder* sub component, which builds a query that targets the fields corresponding to the identified category and entity types.

Next, the *Query expansion* module enriches the query with terms that are likely to occur in the relevant datasets through a strategy that is discussed and evaluated in the next sections. Finally, the query is processed by the *retrieval algorithm* component to obtain datasets related to the query terms, ranked according to their relevance to the query. In this step, we use a standard vector space model (VSM) approach using TF-IDF weighting (54) and cosine similarity as implemented in Lucene (55) (https://lucene.apache.org/core/6_4_0/core/index.html). The top ranked datasets are then formatted for presentation to the user.

## Query expansion

In Information Retrieval, *Query Expansion (QE)* (3,33) enhances a query with additional terms likely to occur in relevant documents. Hence, given a query representation *Q*, QE aims to select an optimal subset *T_k_* of *k* terms, which are relevant to *Q*, then build an expanded query Q′ such that $Q\prime =Q\cup T_{k}$. Below, in Query expansion for biomedical search section, we describe the methods we use for query expansion, and in Utility of query expansion section, we provide an overview of the performance gain that is targeted through query expansion methods.

### Query expansion for biomedical search

We propose to use and evaluate two query expansion methods. We describe them below.

*The Rocchio Algorithm for Relevance Feedback* **:** The Rocchio algorithm (9) is a classic algorithm for relevance feedback used mainly for query expansion. In brief, it provides a method for incorporating relevance feedback information into the vector space model representing a query (10). The underlying theory behind Rocchio is to find a query vector $\vec{Q\prime }$, that maximizes the similarity of the query with relevant documents while minimizing similarity with irrelevant documents. Typically, a pseudo-relevance feedback (PRF) set of *k* top ranked documents obtained after an initial run of the query is considered as the set of relevant documents to build $\vec{Q\prime }$. The formula and variable definitions for Rocchio relevance feedback are shown in the following equation:

(1) $$\vec{Q\prime }=(\alpha \times \vec{Q})+\left(\beta \times \frac{1}{\left|D_{R}\right|}\times \sum_{\vec{d_{r}}\in D_{R}}\vec{d_{r}}\right)-\left(\gamma \times \frac{1}{\left|D_{NR}\right|}\times \sum_{\vec{d_{nr}}\in D_{NR}}\vec{d_{nr}}\right)$$

where $\vec{d_{r}}$ is a relevant document vector, $\vec{d_{nr}}$ is an irrelevant document vector, $\left|D_{R}\right|$ is the set of relevant documents, $\left|D_{NR}\right|$ is the set of irrelevant documents and *α*, *β*, *γ* are, respectively, the weights of the original query, the relevant documents and the irrelevant documents. We have fixed the values of these parameters to: *α* = 1, β=0.5, and γ=0.1 as generally suggested for Rocchio Classification (3). We refer to this method as Rocchio (We used the LucQE module, which provides an implementation of the Rocchio method for Lucene. http://lucene-qe.sourceforge.net/).

*Biomedical Lexicon for Query Expansion* **:** This method is based on a biomedical lexicon. This lexicon is built from (i) gene names extracted from the NCBI gene database (56) (https://www.ncbi.nlm.nih.gov/gene), (ii) organism names extracted from the NCBI taxonomy (56) (https://www.ncbi.nlm.nih.gov/Taxonomy/Browser/wwwtax.cgi) and (iii) disease terms taken from the Unified Medical Language System (UMLS) (57) (https://www.nlm.nih.gov/research/umls/). We use this lexicon in query expansion in a two step process: (1) we match query terms to terms in the lexicon and (2) access the corresponding database record for the matched lexical item to obtain associated terms which are then added to the query. These associated terms include synonyms, acronyms, common denominations, etc. Individual terms are selected for inclusion in the query based on the *idf* score, following the intuition that terms that occur frequently in the collection are of low utility, and terms that occur rarely are of high utility. Then, the top *k* terms are added to the original query in order to enrich it with additional information. We refer to this query expansion method as BioMedLexicon.

### Utility of query expansion

To illustrate the utility of query expansion in the context of biomedical search and in order to provide an insight into upper-bound retrieval performance, we carry out a qrels term selection analysis. Specifically, we enrich the initial query with the top terms obtained by applying the Rocchio formula, given both the relevant and irrelevant datasets provided in the qrels file.

Table 2 provides insight into the utility of query expansion for the Query 1, using different retrieval metrics. When querying only the *title* field of our index, the baseline query, which is the original query (provided in the header row) after stemming and stop-word removal, has an Average Precision (AP) of 0.042 and an inferred normalized discounted cumulative gain (infNDCG) [The infNDCG is a metric that incorporates graded relevance judgments, which is estimated using incomplete relevance judgments (58).] (59,58) of 0.209 (its performance is provided in the footer row). However, by adding the 15 top terms obtained using the Rocchio formula one at a time to the original query, we can measure the new performance values to show the impact of these terms and the effect of query expansion on the retrieval quality. The added terms have been sorted in the order of decreasing infNDCG. We can observe that there are nine terms (highlighted in boldface) that lead to increases in the infNDCG of the original query when they are (individually) added to the original query. For example, the term ‘*CheY’* increases the infNDCG value from 0.209 to 0.585, which represents 179% retrieval improvement. On the other hand, generic terms like ‘*Thermotoga’* decrease significantly the performance of the query (from 0.209 to 0.030). Therefore, the selection of meaningful terms during the query expansion process is critical for increasing the retrieval performance.

**Table 2.** Sample of terms extracted from the qrels and added to the Query 1^a^

Query 1: Find protein sequencing data related to bacterial chemotaxis across all databases				
Term added	P@100	Recall	infAP	AP	infNDCG
*CheY*	**0.840**	**0.177**	**0.112**	**0.136**	**0.585**
*CheA*	**0.460**	**0.103**	**0.040**	**0.057**	**0.412**
*BeF3*	0.370	**0.111**	**0.040**	**0.058**	**0.354**
*Manihotis*	0.410	**0.124**	**0.060**	**0.056**	**0.332**
*MotB*	**0.480**	**0.105**	**0.037**	**0.065**	**0.328**
*Axonopodis*	0.300	**0.128**	**0.046**	**0.045**	**0.325**
*NOX*	0.300	**0.097**	**0.024**	**0.047**	**0.300**
*Structure*	0.310	**0.091**	**0.025**	**0.052**	**0.264**
*Crystal*	0.220	**0.089**	**0.028**	0.039	**0.256**
Complex	0.160	0.086	**0.026**	0.036	0.208
Protein	0.320	**0.087**	**0.027**	**0.059**	0.206
Xanthomonas	0.200	**0.128**	**0.020**	0.019	0.191
Maritima	0.200	**0.102**	0.010	0.030	0.165
Domain	**0.470**	0.081	**0.023**	0.04	0.115
Thermotoga	0.200	**0.102**	0.010	0.030	0.030

***Baseline***	**0.450**	**0.086**	**0.017**	**0.042**	**0.209**

^a^Values in bold are improvements over the baseline.

**Figure 3.**
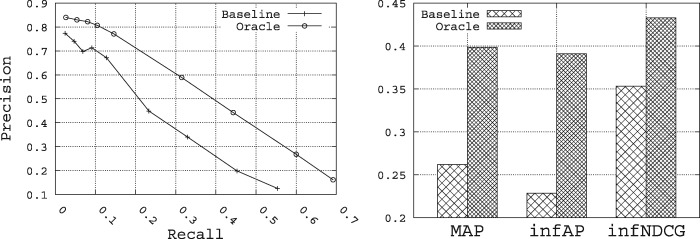
The utility of query expansion for the 15 queries.

Figure 3 shows the summary upper-bound performance for Precision, Recall, MAP, inferred AP (infAP) (60) and infNDCG that can be achieved for the set of 15 queries when querying both the *title* and *description* fields in the index. ‘Baseline’ refers to a run that uses the vector space model (VSM) approach using TF-IDF weighting (54) and cosine similarity using the Lucene search engine (55) and the original query. ‘Oracle’ refers to the situation where the 10 top terms obtained using the Rocchio formula are added to the original query. In this situation, we use both the relevant and irrelevant datasets provided in the qrels file to build the best possible query that pulls the most relevant datasets. This gives us an upper-bound on the performance that can be realized through query expansion for this set of queries when querying only the *title* and *description* fields in the index. It is this statistically significant improvement in performance through query expansion that we intend to target through query expansion.

## Experimental evaluation

In this section, we discuss the retrieval performance of our solution for biomedical search. The configuration options and associated questions that were considered are the following:

1. *Queried fields***:** The datasets of the collections contain several fields that were indexed, i.e. title, description, keywords, organisms, etc. The question that we consider is Are all fields useful for biomedical search? Are certain fields more appropriate to query for certain types of queries?
2. *Query category filter***:** Each dataset is associated to a given category of data, e.g. protein structures for the PDB database. On the other hand, some queries may target a particular type of data, e.g. Question 5 targets gene expression data. Hence, we consider the effectiveness of a category-based filter.
3. *Query expansion source***:** The *title* field, the *description* field, and the biomedical lexicon are different possible sources of terms. We consider what is the best source of expansion terms. For instance, are words in the title of particularly high value as expansion terms? We omit the use of the other fields in the datasets as a source of expansion terms, noting that not all fields are available in all the datasets as shown in Table 1.
4. *Term selection method***:** We consider the two different query expansion methods described above, i.e. Rocchio and BioMedLexicon and ask, what is the best QE method for biomedical search?

### Retrieval performance metrics

To assess the retrieval performance, in addition to the inferred performance measures of the official challenge, we also consider standard IR measures including Precision, Recall and Mean Average Precision (MAP). These are the most commonly used, and well understood, performance measures in IR. Moreover, as the number of datasets judged per query ranges from 836 to 1734, the relevance judgments are not particularly sparse, and hence Precision, Recall and MAP can be consistent metrics. The use of inferred measures does not seem necessary given these sufficient numbers of judgments (58,60).

### Analysis of the index fields

Before discussing the effectiveness of the multi-field query strategy that we propose, we first analyze the impact of querying the different fields of the index we have built. Table 4 shows the retrieval performance we obtain by querying one field of the index, or two fields simultaneously. We only show the possible combinations of querying two fields, as showing the results of querying more than two fields requires to draw a complex table of multiple dimensions.

**Table 4.** Retrieval performance summary of the baseline^a^

Metric	Queried fields	
	Description	Title and description
	Baseline 1	Baseline 2
**Precision@10**	0.6067	**0.7467**
**MAP**	0.2171	**0.2669**
**infAP**	0.2088	**0.2348**
**infNDCG**	**0.3609**	0.3575

^a^Values in bold are improvements over the baseline.

The main diagonal of Table 4 represents a search conducted over only the record field in the corresponding row/column. For example, the values given in the first line and first column represent the retrieval performance obtained when querying only the *title* field of each record, the values in the second line and the second column are those obtained when querying only the *description* field, and so on. The values off the diagonal in Table 4 represent the retrieval performance obtained when querying two fields simultaneously. For example, the values given in the first line and second column represent the retrieval performance obtained when querying both the *title* field and the *description* field, the values in the first line and the third column are those obtained when querying both the *title* field and the *keywords* field, etc.

Overall, several lessons can be drawn from Table 4:

1. Among all fields, the *title* and the *description* provide the best retrieval performance. This is quite expected since these two fields are the most common across the repositories (see Table 1).
2. Even if the *keywords* field is available in several repositories which contain many relevant datasets, it seems to provide poor retrieval performance. Also, when combined with the *title* or the *description* fields, the *keywords* field drastically decreases the retrieval performance.
3. Querying the *organisms* field alone, which is the third most common field for all the repositories, leads to very poor performance (0.01% MAP and 0.7% infNDCG). This is mainly due to two reasons: first, as shown in Table A5, not all queries mention organisms, and second, the repositories are biased toward the most popular organisms (https://www.ncbi.nlm.nih.gov/Taxonomy/Browser/wwwtax.cgi).
4. Querying both the *description* field and the *organisms* field slightly improve the performance, as the P@10 increases from 0.6 to 0.633, the MAP increases from 0.2165 to 0.2362, and the infAP increases from 0.2075 to 0.2276.
5. All other fields seem to have little value for retrieval, since when queried alone or combined with other fields, the retrieval performance sometimes decrease or increase insignificantly. Again, this is due to the fact that most of them are not common to all repositories as shown in Table A5.

At this point, we consider the following question: ‘*Can we use the specific fields* (i.e. *genes, PMID, diseases, and treatment, etc.) to effectively improve the retrieval performance?’* To answer this question, we carry out a per field analysis in what follows.

#### Literature-based fields analysis

Since the literature-based fields (*Art. Title* and *Art. Abstract*) are only available into the *PDB*, *YPED*, and *PeptideAtlas* repositories, we shortened the list of queries to assess (in this subsection) to those for which there exist several relevant datasets in these repositories. This is done to highlight the impact of the literature-based fields on the retrieval performance. This list includes seven queries: Query 1, Query 2, Query 3, Query 6, Query 8, Query 11 and Query 13 (see Table A5).

**Figure 4.**
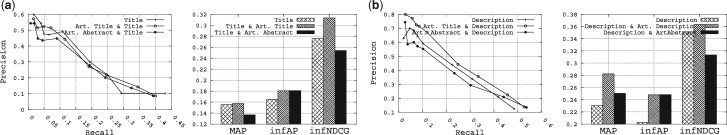
The utility of the literature-based fields.

Figure 4 shows the results obtained for this analysis. Specifically, Figure 4a shows the results obtained when querying the *title* field combined with the literature-based fields, and Figure 4b shows the results obtained when querying the *description* field combined with the literature-based fields. Overall, when combined with either the *title* or the *description* fields, the literature-based fields significantly improve the retrieval performance for MAP, infAP and infNDCG. For example, comparing querying the *description* field alone and the *description* field plus the title of the research articles field, the MAP is boosted from 0.2304 to 0.2824 (22.56% improvement), and the infAP is boosted from 0.2025 to 0.248 (22.46% improvement). However, regarding the Precision/Recall curves, the improvement is not really clear.

Finally, we conclude here by saying that the literature is clearly a useful source of information for retrieval by providing a detailed description of a given dataset. The literature has also proven its utility in other contexts including data quality assessment (61,62,63). This should encourage research centers to link the research literature to their datasets.

#### Gene field analysis

Since only a few queries mention genes (see Table A5), we again shortened the list of queries to assess (in this subsection). This is done to focus on the impact of the *gene* fields on the retrieval performance. This list includes eight queries: Query 2, Query 3, Query 5, Query 6, Query 9, Query 11, Query 13 and Query 15.

**Figure 5.**
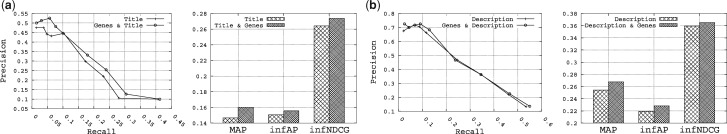
The utility of the Gene fields on the queries that mention genes.

Figure 5 shows the results obtained for this analysis. Specifically, Figure 5a shows the results obtained when querying the *title* field combined with the *gene* field, and Figure 5b shows the results obtained when querying the *description* field combined with the *gene* field. Overall, when combined with either the *title* or the *description* fields, the *gene* field significantly improves the retrieval performance for all metrics (Precision, Recall, MAP, infAP and infNDCG). For example, comparing querying the *title* field alone and the *title* field plus the *gene* field, the MAP is boosted from 0.1467 to 0.1601 (9.13% improvement), and the infNDCG is boosted from 0.2641 to 0.2737 (3.63% improvement). Therefore, given the results shown, it is clear that querying the *gene* field for queries that mention genes is an effective strategy, which results in improved retrieval performance.

#### Disease-based fields analysis

**Figure 6.**
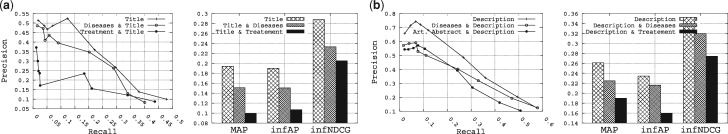
The utility of the disease-based fields on the queries that mention diseases.

The disease-based fields include both the *diseases* field and the *treatment* field. Again, to effectively study the utility of these two fields, we have shortened the list of queries to those that are mentioning diseases (see Table A5). This list includes seven queries: Query 2, Query 4, Query 6, Query 11, Query 12, Query 13 and Query 15. The results obtained are shown in Figure 6. Specifically, Figure 6a shows the results obtained when querying the *title* field combined with the disease-based fields, and Figure 6b shows the results obtained when querying the *description* field combined with the disease-based fields.

At first glance, the retrieval performance drastically decreases when associating the disease-based fields with the *title* or *description* fields. After a brief failure analysis, we believe that this is mainly due to three reasons: (i) among the three repositories that provide the *diseases* field, roughly 30% of their datasets donas contain any information in this field; (ii) among the three repositories that provide the *treatment* field, roughly 62% of their datasets donas contain any information in this field; and (iii) for the *diseases* field, usually general terms like ‘cancer’ or ‘tumor’ are used in the datasets, which may bias the search. Therefore, the disease-based fields are not helpful for retrieval since they contribute to drastically reducing the retrieval performance.

#### Category-based filter analysis

Another field that is indexed but we have not used for retrieval in our experiments is the *category* field. This information may be useful as a filter for selecting certain types of data. Indeed, as the dataset collection is an aggregation of various types of biomedical data, queries may target a specific kind of dataset. The *Query expansion & Modification* module is responsible for guessing what type of data are targeted by identifying specific keywords in the query in order to target the corresponding repositories. This list of keywords includes: gene expression, clinical trials, protein structure, etc.

**Figure 7.**
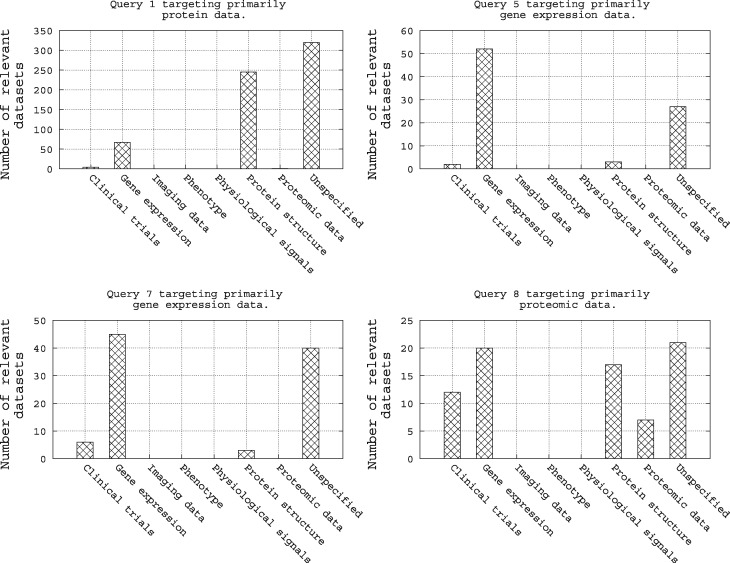
Analysis of the query qrels with respect to the repository categories for Query 1, Query 5, Query 7 and Query 8.

As shown in Table A5, Query 1, Query 5, Query 7 and Query 8 target, respectively, protein data, gene expression data, again gene expression data and proteomic data. Hence, intuitively, we can imagine restricting a query to target its specific category of biomedical data through a filter. To assess this simple intuition, we refer to Figure 7. Here we show an analysis of these four queries with respect to the categories of their relevant datasets as provided in the qrels file. There are two notable trends in this figure: (i) first, the four queries have a high number of relevant datasets in the category of biomedical data they are targeting, e.g. Query 1, which targets protein data, has a high number of relevant datasets in repositories of gene expression and protein structure, and (ii) second, for the four queries, there are a high number of relevant datasets in the ‘unspecified’ repository category. Hence, it is clear that when the category of a repository is ‘unspecified’, the repository should always be targeted.

**Figure 8.**
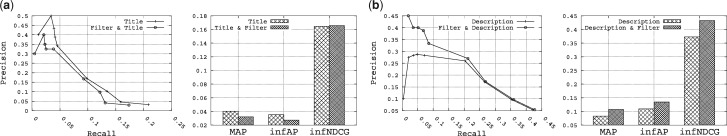
The utility of the category-based filter on the queries that mention specific type of biomedical data.

The effect of this category-based filter is shown in Figure 8 for the four queries that mention specific types of biomedical data. Specifically, Figure 8a shows the results obtained when querying the *title* field combined with the *category-based* filter, and Figure 8b shows the results obtained when querying the *description* field combined with the *category-based* filter. The results obtained show that when querying the *title* field only, applying the category filter has no impact or may slightly decrease the retrieval performance. However, when querying the *description* field and applying the category filter, the retrieval performance is considerably improved, boosting the MAP from 0.0826 to 0.1074, infAP from 0.1097 to 0.1345, and infNDCG from 0.3727 to 0.4325. For infNDCG, this represents an improvement of 16.04%. Hence, a category-based filter can improve the retrieval performance in this context of biomedical data search.

### Choosing the baseline

For our comparison baseline, we chose the configuration that gives the best retrieval performance from Table 3. It consists in a straightforward approach, in which we query only the most common fields in our index, namely the *title* and *description* fields. This baseline retrieves datasets that contain the query terms in their title or description. We believe this is a reasonable approach as it ensures a search for relevant datasets in almost all the repositories, given that these two fields are common to all repositories (except *NeuroMorpho*, *CIA* (64) (The Cancer Imaging Archive is a large archive of medical images of cancer accessible for public download.), and *ProteomeXchange* (65) (The ProteomeXchange consortium provides a single point of submission of MS proteomics data to the main existing proteomics repositories.) where the datasets do not contain the *description* field).

**Table 3.** Multi-fields query retrieval performance when querying one field (on the diagonal) or two fields simultaneously (off the diagonal) on the set of 15 bioCADDIE queries^a^

		Fields									
		Title	Description	Keywords	Organisms	Art. Title	Art. Abstract	Genes	Diseases	Treatment	Metric
Fields	Title	0.5667	**0.7467**	0.5200	0.4400	0.3667	0.3933	0.5600	0.4000	0.2067	**P@10**
		0.1509	**0.2669**	0.0982	0.1283	0.1108	0.0864	0.1543	0.0995	0.0644	**MAP**
		0.1438	**0.2348**	0.0999	0.1088	0.1136	0.1024	0.1461	0.0903	0.0624	**infAP**
		0.2615	0.3575	0.2361	0.2438	0.2346	0.1977	0.2609	0.1778	0.1510	**infNDCG**
	Description	–	0.6067	0.5733	0.6333	0.6867	0.4933	0.6067	0.4867	0.4133	**P@10**
		–	0.2171	0.1792	0.2362	0.2297	0.2034	0.2171	0.1676	0.1422	**MAP**
		–	0.2088	0.1822	0.2276	0.2171	0.2074	0.2083	0.1688	0.1306	**infAP**
		–	**0.3609**	0.3241	0.3525	0.3607	0.3191	0.3604	0.3098	0.2702	**infNDCG**
	Keywords	–	–	0.2267	0.2667	0.3067	0.3533	0.3133	0.2600	0.1533	**P@10**
		–	–	0.0322	0.0321	0.0314	0.0343	0.0344	0.0316	0.0168	**MAP**
		–	–	0.0407	0.0391	0.0447	0.0469	0.0437	0.0397	0.0256	**infAP**
		–	–	0.1462	0.1470	0.2207	0.1627	0.1404	0.1246	0.0996	**infNDCG**
	Organisms	–	–	–	0.0067	0.1467	0.2000	0.1867	0.1267	0.0400	**P@10**
		–	–	–	0.0001	0.0081	0.0123	0.0071	0.0145	0.0007	**MAP**
		–	–	–	0.0001	0.0130	0.0211	0.0072	0.0136	0.0011	**infAP**
		–	–	–	0.0076	0.1598	0.0923	0.0163	0.0439	0.0099	**infNDCG**
	Art. Title	–	–	–	–	0.1733	0.2133	0.2400	0.2267	0.1267	**P@10**
		–	–	–	–	0.0113	0.0164	0.0153	0.0258	0.0057	**MAP**
		–	–	–	–	0.0230	0.0360	0.0237	0.0346	0.0092	**infAP**
		–	–	–	–	0.1136	0.1188	0.1189	0.1125	0.0545	**infNDCG**
	Art. Abstract	–	–	–	–	–	0.1667	0.2800	0.2133	0.2067	**P@10**
		–	–	–	–	–	0.0149	0.0201	0.0263	0.0125	**MAP**
		–	–	–	–	–	0.0266	0.0294	0.0362	0.0197	**infAP**
		–	–	–	–	–	0.1035	0.1053	0.1033	0.0906	**infNDCG**
	Genes	–	–	–	–	–	–	0.1933	0.1600	0.0467	**P@10**
		–	–	–	–	–	–	0.0088	0.0175	0.0015	**MAP**
		–	–	–	–	–	–	0.0091	0.0165	0.0025	**infAP**
		–	–	–	–	–	–	0.0096	0.0459	0.0148	**infNDCG**
	Diseases	–	–	–	–	–	–	–	0.1200	0.1000	**P@10**
		–	–	–	–	–	–	–	0.0152	0.0117	**MAP**
		–	–	–	–	–	–	–	0.0140	0.0119	**infAP**
		–	–	–	–	–	–	–	0.0394	0.0436	**infNDCG**
	Treatment	–	–	–	–	–	–	–	–	0.0333	**P@10**
		–	–	–	–	–	–	–	–	0.0006	**MAP**
		–	–	–	–	–	–	–	–	0.0012	**infAP**
		–	–	–	–	–	–	–	–	0.0099	**infNDCG**

^a^Values in bold are improvements over the baseline. (–) is used to avoid duplicating the results as the table is symmetric.

Table 4 summarizes the performance of our simple strategy when querying the *description* field (Baseline 1), and both the title and the description fields at a time (Baseline 2). Although Baseline 1 slightly outperforms Baseline 2 in term of infNDCG, Baseline 2 shows significant improvement for the other metrics. However, due to this higher value obtained for infNDCG by Baseline 1, we have included it for comparison. Overall, we have selected strong baselines, which will be used in evaluating the performance of our proposed solution in the remainder of the paper.

### Multi-field query builder analysis

The results obtained in the previous subsection gave us the intuition that led us to propose our strategy for a multi-field query builder. Recall that the query is processed by three sub-components, *DNorm*, *GenNorm* and *Linnaeus*, which, respectively, identify diseases, genes and organisms mentioned in the query. Once done, a new query is built as follows:

1. The *title*, *description* and *article title* fields are always targeted with the full set of query terms.
2. If organisms are identified in the query, the *organism* field is targeted with the organism names.
3. If genes are identified in the query, the emphgene field is targeted with the gene names.

**Figure 9.**
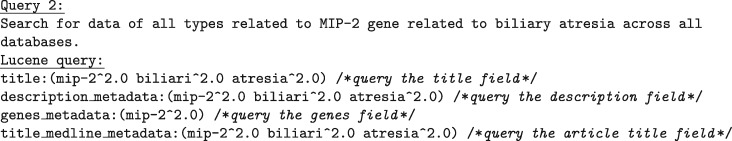
Example of Query 2 transformed into the Lucene query syntax targeting multiple fields. Note that concept terms identified in the query are boosted with a factor of 2.

In addition, we also boost the concepts identified in the query by a factor of 2 in order to emphasize these concepts in the datasets. This parameter value was adjusted upon the training set given for the challenge. Figure 9 shows the example of Query 2 transformed into a Lucene query syntax (https://lucene.apache.org/core/6_4_1/queryparser/org/apache/lucene/queryparser/classic/package-summary.html) by the multi-field query builder.

**Figure 10.**
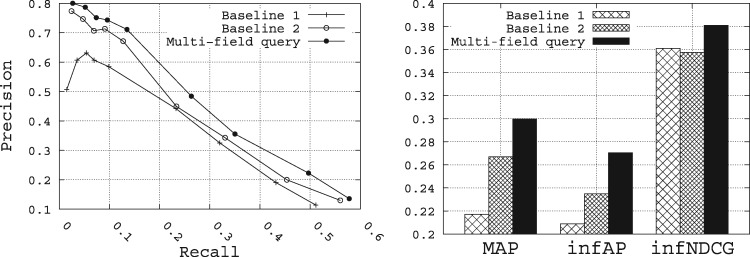
Performance of the multi-field query method with respect to the baselines.

Figure 10 shows the retrieval performance of the multi-field query builder compared with the two baselines. At first glance, clearly the multi-field approach outperforms the two baselines for all the metrics. Compared with Baselines 1 and 2, MAP is improved from 0.2171 and 0.2669, respectively, to 0.2996, infAP is improved from 0.2088 and 0.2348, respectively, to 0.2703, and infNDCG is improved from 0.3609 and 0.3575, respectively, to 0.3809. The results obtained here clearly demonstrate that a multi-field query approach, which identifies concepts and queries specifically the relevant fields for those concepts, is an effective approach for biomedical data search. In this way, genes identified in a query are used to query the *gene* field, organisms identified in a query are used to query the *organisms* field, and so on.

### Query expansion results

**Figure 11.**
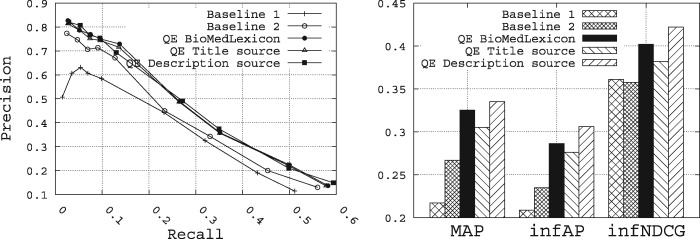
Performance of the query expansion methods compared with the baselines.

To summarize all the results obtained over all the possible configurations for the query expansion, Figure 11 shows the retrieval performance obtained for all the QE methods, while enriching the initial queries with the top five terms returned for each method. Selecting the top five terms to enrich the initial queries is not the optimal number that maximizes the retrieval performance, but we believe it is a reasonable number that maintains the queryai original sense. From these results, we make the following observations:

- The best query expansion method is the Rocchio method while using the description field as source of query expansion, followed by the query expansion method based on the biomedical lexicon, and then the Rocchio method while using the *title* field as source of query expansion.
- The description field contains terms of high quality for query expansion compared with those of the *title* field.
- The three query expansion methods allow us to outperform the two baselines.

Overall, the Rocchio method using the *description* field as source of query expansion allows us to improve the MAP for Baselines 1 and 2 from 0.2171 and 0.2669, respectively, to 0.3351, the infAP for Baselines 1 and 2 from 0.2088 and 0.2348, respectively, to 0.3062, and the infNDCG from 0.3609 and 0.3575, respectively, to 0.4219.

Comparing the two methods of query expansion, where the QE method based on the *description* field as source of terms slightly outperforms the one based on the biomedical lexicon as source of terms (roughly a marginal improvement of 3%, 7% and 5% for, respectively, MAP, infAP and infNDCG), we believe it is mainly due to the fact that overall, we observe a high value for P@10 (roughly 0.78 in Figure 10). Indeed, as the Rocchio query expansion method is based on a pseudo-relevance feedback set of the top-*k* ranked datasets obtained after an initial run of the query (we have chosen *k* = 10 as this value has proven to be reasonable in other applications), this set is likely to contain roughly 7 out of 10 relevant datasets. Hence, the Rocchio formula is likely to expand the query with terms that are likely to be common to the other relevant datasets. This may partly explain the superiority of this approach.

On the other hand, despite the fact that the query expansion method based on the biomedical lexicon is providing decent retrieval results, it is much less computationally intensive since it does not require to compute any relevance feedback set or to make any initial execution of the query. Therefore, in terms of trade-off between efficiency, execution time and retrieval accuracy, the query expansion method based on the biomedical lexicon offers the best performance for a prototype biomedical data search engine that is devoted to be used at a large scale.

## Discussion of the results of the bioCADDIE dataset retrieval challenge

In the bioCADDIE Dataset Retrieval Challenge 2016, 10 participants submitted 45 runs (maximum five runs per team) for the official evaluation (2) (https://docs.google.com/spreadsheets/d/1z9v053gC7CBhtKYEWP-ZLglAROBMwyjp5ellTZ1qZnk/). Our team named BioMelb submitted the following five runs:

- *Run* 1**:** The first run has been used as a baseline, where we only queried the repository, the title and the description fields of the index. This is similar to Baseline 2 that we have described.
- *Run* 2**:** In the second run, we simply queried the following fields of the index using the specific terms identified by the parser: the repository, the title, the description, the keywords, the genes, the diseases and the organisms.
- *Run* 3**:** In the third run, we used exactly the same queries strategy as in Run 2, except that we also queried the Titles of the literature extracted from Medline.
- *Run* 4**:** In the fourth run, we used exactly the same queries strategy as in Run 3, except that we also queried the Abstracts of the literature extracted from Medline.
- *Run* 5**:** In the fifth run, we used a query expansion strategy. We used the same strategy as in Run 1, except that we manually expanded some query terms with synonyms or associated terms, extracted mainly from Wikipedia and the NCBI taxonomy. This includes expanding gene names, diseases names and organism names with related terms.

In terms of comparison of official runs with what has been presented in this paper, Run 1 corresponds to the Baseline 2 that we have described, and Run 3 uses the multi-field query we have described. Run 5 is similar to the multi-field query expansion using the biomedical lexicon we have presented above; the only difference is that during the official challenge, Run 5 utilized a manual query building strategy, whereas in this paper, we have described an automated version of that strategy.

The submitted runs were formally evaluated using the following four IR metrics: infAP, infNDCG, NDCG@10 and P@10, while emphasis was placed on the infNDCG metric. The infNDCG values of the submitted runs ranged from 0.2539 to 0.5132, and the P@10 values ranged from 0.1467 to 0.8267.

Considering the infNDCG metric, the UCSD team’s method achieved the best performance with an infNDCG of 0.5132, followed by the UIUC GSIS teamow method with an infNDCG of 0.4502 which represents a big gap (an improvement of 14% in a relative term). Then, the other teams obtained infNDCG values that are close to each other, where our team BioMelb ranked seventh with Run 4 which achieved an infNDCG of 0.4017.

As for the P@10 metric, the Elsevier team’s method achieved the best performance with a value of 0.8267, followed by our teamow Run 5 with a P@10 value of 0.7733. Hence, the method proposed in this paper achieved the second best result in terms of precision compared with the other competitors.

At this point, comparing all runs based on the infNDCG and P@10, it is interesting to notice that the run that achieved the best performance in term of infNDCG would be ranked 9th in the ranking based on P@10. The same observation is made for the second best run based on infNDCG, which would also be ranked nineth in the ranking based on P@10. This clearly indicates that the infNDCG metric is not always correlated with the P@10 metric. Furthermore, it is interesting to notice that the infNDCG metric is also not correlated with the classic NDCG metric. Indeed, the run that allowed the UCSD team to be ranked first in term of infNDCG would rank the UCSD team ninth in the ranking based on the classic NDCG metric. In term of NDCG, our best run is ranked second.

Last but not least, it is important to note that the bioCADDIE test query set is extremely small, with just 15 queries. Typical IR challenges such as the TREC challenges include at least 50 test queries (66–68). This may be considered to be a low number of test queries for providing consistent and robust evaluation results. We believe that this explains why the performance based on the inferred metrics does not correlate with the performance based on the conventional metrics across all participants (e.g. the UIUC-GSIS team ranks second in term of inNDCG but 10th in term of NDCG). Indeed, the robustness and consistency of most of the inferred measures with respect to conventional measures has only been demonstrated on a larger number of queries (58–60,69). As discussed in Retrieval performance metrics section, this also drives our choice to present our results in terms of the conventional metrics.

## Conclusion

In this paper, we presented a method for retrieving and searching biomedical data through query formulation. In particular, the method proposed transforms the initial query into a multi-field query that is then enriched with terms that are likely to occur in the relevant datasets, using query expansion. We compared and evaluated two query expansion strategies, one based on the Rocchio method and another based on a biomedical lexicon. We performed a comprehensive comparative evaluation of our method on the bioCADDIE dataset collection for biomedical retrieval.

We have demonstrated the effectiveness of our multi-field query building method compared to baseline methods which consist of a straightforward approach of querying the *title* and/or the *description* fields (for Baselines 1 and 2, MAP is improved from 0.2171 and 0.2669, respectively, to 0.2996, infAP is improved from 0.2088 and 0.2348, respectively, to 0.2703, and infNDCG is improved from 0.3609 and 0.3575, respectively, to 0.3809.). We have also shown the benefits of query expansion, where the Rocchio method using the *description* field as source of query expansion allows to improve the MAP for Baselines 1 and 2 from 0.2171 and 0.2669, respectively, to 0.3351, the infAP for Baselines 1 and 2 from 0.2088 and 0.2348, respectively, to 0.3062, and the infNDCG from 0.3609 and 0.3575, respectively, to 0.4219.

We observed that the Rocchio QE method based on the *description* field as source of terms slightly outperforms the approach based on the biomedical lexicon as source of terms (roughly a marginal improvement of 3%, 7% and 5% for, respectively, MAP, infAP and infNDCG). The Rocchio query expansion method expands the query with terms that are likely to occur in relevant datasets, as evidence by high value for P@10 obtained. We also highlighted the fact that the query expansion method based on the biomedical lexicon is providing decent retrieval results, but is computationally much lighter since it doesner require computation of any relevance feedback set or any initial execution of the query. Hence, in terms of trade-off between efficiency, execution time and retrieval accuracy, the query expansion method based on the biomedical lexicon offers strong performance and should be considered for a biomedical data search engine to be used at a large scale.

In conclusion, although our approach is ranked seventh in term of infNDCG in the official bioCADDIE results, it ranks second in term of P@10 and NDCG. Hence, we believe that the proposed method provides good retrieval performance and warrants further exploration of query expansion strategies in this context. For instance, we plan to explore a hybrid approach based on Rocchio and the biomedical lexicon methods presented in this paper. Consequently, the conclusions and observations drawn in this paper should be benefit for the development of a Data Discovery Index prototype or the improvement of the existing one (DataMed).

Finally, although the methods in this work were developed in the particular context of the DataMed project, the conclusions derived from our investigation may have broader application for search over repositories containing diverse data collections. Indeed, depending on the nature of a given query, the type of targeted data and the set of targeted repositories, the query can be reformulated following the methodology suggested in this paper to better take advantage of the semantic structure of the query. In particular, the insight that different fields may contribute differently to retrieval and query expansion performance is likely to generalize. Future work includes the exploration of re-ranking and the improvement of IR model using external sources of data such as the literature (70,71).

## Funding

The project received funding from the Australian Research Council through a Discovery Project grant, DP150101550. The bioCADDIE Dataset Retrieval Challenge was supported by NIH grant U24AI117966.

*Conflict of interest*. None declared.

## Figures and Tables

**Table A5.** Details of the queries

Queries		Organisms	Genes	Diseases	Category
Query 1:	Find protein sequencing data related to bacterial chemotaxis across all databases	—	—	—	+
Query 2:	Search for data of all types related to MIP-2 gene related to biliary atresia across all databases	—	+	+	—
Query 3:	Search for all data types related to gene TP53INP1 in relation to p53 activation across all databases	—	+	—	—
Query 4:	Find all data types related to inflammation during oxidative stress in human hepatic cells across all databases	+	—	+	—
Query 5:	Search for gene expression and genetic deletion data that mention CD69 in memory augmentation studies across all databases	—	+	—	+
Query 6:	Search for data of all types related to the LDLR gene related to cardiovascular disease across all databases	—	+	+	—
Query 7:	Search for gene expression datasets on photo transduction and regulation of calcium in blind *Drosophila melanogaster*	+	—	—	+
Query 8:	Search for proteomic data related to regulation of calcium in blind *D. melanogaster*	+	—	—	+
Query 9:	Search for data of all types related to the ob gene in obese *Mus musculus* across all databases	+	+	—	—
Query 10:	Search for data of all types related to energy metabolism in obese *M. musculus*	+	—	—	—
Query 11:	Search for all data for the HTT gene related to Huntingtoner disease across all databases	—	+	+	—
Query 12:	Search for data on neural brain tissue in transgenic mice related to Huntingtoner disease	+	—	+	—
Query 13:	Search for all data on the SNCA gene related to Parkinsonne disease across all databases	—	+	+	—
Query 14:	Search for data on nerve cells in the substantia nigra in mice across all databases	+	—	—	—
Query 15:	Find data on the NF-kB signaling pathway in *Myasthenia gravis* (MG) patients	—	+	+	—

(x) means the concept is present in the query, (—) means the concept is not present in the query. These marks do not imply any ‘positive’ or ‘negative’ information except for the presence or the absence of the considered concept in the query.

## Appendix

The list of the queries given as test for the bioCADDIE challenge is given inTable A5.
